# Molecular evolution and SSRs analysis based on the chloroplast genome of *Callitropsis funebris*

**DOI:** 10.1002/ece3.7381

**Published:** 2021-03-20

**Authors:** Jingyao Ping, Peipei Feng, Jinye Li, Rongjing Zhang, Yingjuan Su, Ting Wang

**Affiliations:** ^1^ College of Life Sciences South China Agricultural University Guangzhou China; ^2^ School of Life Sciences Sun Yat‐sen University Guangzhou China; ^3^ Research Institute of Sun Yat‐sen University in Shenzhen Shenzhen China

**Keywords:** adaptive evolution, *Callitropsis funebris*, chloroplast genome, phylogeny, SSRs

## Abstract

Chloroplast genome sequences have been used to understand evolutionary events and to infer efficiently phylogenetic relationships. *Callitropsis funebris* (Cupressaceae) is an endemic species in China. Its phylogenetic position is controversial due to morphological characters similar to those of *Cupressus*, *Callitropsis,* and *Chamaecyparis*. This study used next‐generation sequencing technology to sequence the complete chloroplast genome of *Ca. funebris* and then constructed the phylogenetic relationship between *Ca. funebris* and its related species based on a variety of data sets and methods. Simple sequence repeats (SSRs) and adaptive evolution analysis were also conducted. Our results showed that the monophyletic branch consisting of *Ca. funebris* and *Cupressus tonkinensis* is a sister to *Cupressus,* while *Callitropsis* is not monophyletic; *Ca. nootkatensis* and *Ca. vietnamensis* are nested in turn at the base of the monophyletic group *Hesperocyparis*. The statistical results of SSRs supported the closest relationship between *Ca. funebris* and *Cupressus*. By performing adaptive evolution analysis under the phylogenetic background of Cupressales, the Branch model detected three genes and the Site model detected 10 genes under positive selection; and the Branch‐Site model uncovered that *rpo*A has experienced positive selection in the *Ca. funebries* branch. Molecular analysis from the chloroplast genome highly supported that *Ca. funebris* is at the base of *Cupressus*. Of note, SSR features were found to be able to shed some light on phylogenetic relationships. In short, this chloroplast genomic study has provided new insights into the phylogeny of *Ca. funebris* and revealed multiple chloroplast genes possibly undergoing adaptive evolution.

## Graphical abstract

The complete chloroplast genome sequence of *Callitropsis funebris* was obtained for the first time. Phylogenetic tree construction based on various methods and simple sequence repeat characteristic analysis showed that *Ca. funebri* *s* was located at the base of *Cupressus*. In the process of Cupressales evolution, positive selection branches were detected in 3 genes, positive selection sites were detected in 10 genes, and positive selection sites were detected in *rpo*A gene in *Ca. funebris*.

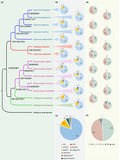

## 1 │ INTRODUCTION

Chloroplast is a unique organelle of green plants and has an independent genetic system. With the development of sequencing technology, chloroplast genome data can be obtained relatively easily, and the massive emergence of chloroplast genome data provides sufficient resources for the study of chloroplast genomics (Tonti‐Filippini et al., 2017). The size of a typical terrestrial plant chloroplast genome is generally 120–160 kb, with a quadripartite structure involving two inverted repeat (IR) sequences that separate the rest of the genome into large and small single‐copy regions (Kwon et al., 2020; Wicke et al., 2011). In particular, conifers are one of the few groups that have lost the canonical IR region (Guo et al., 2014; Kwon et al., 2020; Li et al., 2016; Ping et al., 2020). Compared with nuclear genome and mitochondrion genome, chloroplast genome is characterized by small genome size, high copy number, conservative structure, and moderate nucleotide evolution rate, which has been widely used to understand evolutionary events and to infer efficiently phylogenetic relationships (Barrett et al., 2016; Pacheco et al., 2019; Saina et al., 2018). Similarly, the conservation of chloroplast genes and chloroplast genome structure, gene loss and pseudonymization, gene transfer events, plastid rearrangement, and RNA editing sites make it possible to characterize specific lineages (Bock, 2017; De Santana Lopes et al., 2019; Martin et al., 2014; Vieira et al., 2016).

The repeat sequence, gene sequence, gene content, and GC content of the chloroplast genome can provide more information for phylogeny. Simple sequence repeats (SSRs), also known as microsatellite, are a piece of DNA composed of basic units of 1 to 6 nucleotides that are repeated many times, and it has polymorphism rate at the species level (Cavalier‐Smith, 2002). They are widely distributed in different locations in the genome and are generally <200 bp in length. SSR molecular markers are widely used because of their advantages of codominance, high information content, wide coverage, and easy operation. The SSRs of chloroplast genomes of plants contain rich information on genetic variation and have been widely used in crop species identification, genetic diversity, and relationship studies (Chmielewski et al., 2015) and have a broad prospect of application in plant genetics and taxonomy (Jiménez, 2010).

Chloroplasts, as organelles for plant photosynthesis, are closely related to the adaptation of plants to various environments. Some genes that play a key role in photosynthesis may undergo adaptive evolution along with species radiation (Hasegawa et al., 2009; Zhang et al., 2018). Studies have reported that *clp*P (Erixon & Oxelman, 2008), *rbc*L (Kapralov & Filatov, 2007), *mat*K (Hao et al., 2010), and other chloroplast genes have undergone positive selection. Analysis of adaptive evolution at the genetic level and identification of the key functional sites and amino acid structure can provide evidence for exploring the mysteries of plant evolution, as well as a deeper understanding of genetic structure and function variations (Nei & Kunar, 2000).

Gymnosperms originated more than 300 million years ago and have about 860 species. It includes four of the five main lineages of seed plants: cycads, ginkgos, gnetophytes, and conifers. There are 227 gymnosperm species in China, covering 8 families and 37 genera (Wang et al., 2015). Due to the large genome size, high heterozygosity, and long generation time of populations, understanding the evolution of gymnosperms still faces great difficulties in the era of genomics (Wang & Ran, 2014). At the beginning of the 21st century, *Cupressus* was a hot topic in the conifer systematics, and various suggestions have been proposed to subdivide it into multiple genera (Adams et al., 2009; de Laubenfels, 2009; Farjon et al., 2002; Little, 2006; Mao et al., 2010; Terry et al., 2012). Now cypresses are divided into two groups: Old World cypresses (only *Cupressus*) and New World cypresses (*Cupressus*, *Callitropsis,* and *Hesperocyparis*). There are about 25 species of Old World cypresses, among which *Callitropsis funebris* is endemic to China. It is widely distributed, with rapid growth, strong adaptability, and other characteristics. As a precious wood species, it also has medicinal value and is of very important economic values.

The scaly branchlets of *Ca. funebris* are flat, drooping, with smaller cones and a fewer number of seeds per scale (only 5–6). These characteristics are similar to those of *Chamaecyparis* and *Callitropsis*; but the branchlets of *Ca. funebris* are drooping and homomorphic, unlike those of *Chamaecyparis*, which are flattened and heteromorphic. The cones of *Ca. funebris* mature in the second year and have 3–4 pairs of seed scales with 4 pollen sacs in each scale that are similar to those of *Cupressus*. Zheng and Fu (1978) believed that the main morphological traits of *Ca. funebris* were consistent with those of *Cupressus*, and it was more natural to put it into *Cupressus*. Similarly, Rushforth et al. (2003) divided *Ca. funebris* into Old World cypresses based on molecular trait analysis. The relationship between *Ca. funebris* and *Chamaecyparis* has also been controversial (Farjon, 2005; Gadek & Quinn, 1987; Jagel & Stutzel, 2001). More recently, De Laubenfels et al. (2012) further tried to solve the problems existing in New World cypresses and Old World cypresses according to morphological characteristics, believing that *Callitropsis nootkatensis*, *Xanthocyparis vietnamensis,* and *Cupressus funebris* belong to *Callitropsis*. However, morphological analysis is often limited in the construction of phylogenetic relationships, and the application of molecular data can provide more information for phylogenetic development.

Due to the unique characteristics of *Ca. funebris* and its controversial phylogenetic location, we start from the chloroplast genome to explore its phylogenetic location and further understand related evolutionary events. So, in this study, the complete chloroplast genome of *Ca. funebris* was sequenced using next‐generation sequencing technology. Phylogenetic relationship of Cupressales was constructed based on a variety of data sets and methods. We also analyzed and compared the chloroplast genome characteristics of cypresses (focus on SSRs) and carried out adaptive evolution analysis under the phylogenetic background of Cupressales.

## 2 │ MATERIALS AND METHODS

### 2.1 │ Sampling

Fresh leaves from *Ca. funebris* were sampled from the campus of South China Agricultural University (E113°35', N23°15'). The leaves were wrapped in tinfoil and stored in the liquid nitrogen at −80℃ for later use.

### 2.2 │ DNA extraction and sequencing assembly

The chloroplast genomic DNA was extracted using a new DNAsecure plant genomic DNA extraction kit (TIANGEN), and the DNA library (300 bp) was constructed after fragmentation. Illumina HiSeq2500 platform was used to carry out two‐end sequencing with 150 bp reading length. In order to ensure the quality, the original data must be filtered to remove sequences with joints and low quality. Trimmomatic v0.32 (Bolger et al., 2014) was used to filter data to obtain clean reads. Clean data were spliced and assembled using velvet v1.2.03 (Zerbino & Birney, 2008). DOGMA software (Wyman et al., 2004) was used to predict gene and gene function annotation, and rpsblast v2.2.30+ (Altschul et al., 1997) was used to predict gene COG functional information. Chloroplast genome mapping was performed by OGDRAW v1.3 (https://chlorobox.mpimp‐golm.mpg.de/OGDraw.html) (Greiner et al., 2019). Sequence was submitted through the Banklt platform (https://www.ncbi.nlm.nih.gov/WebSub/).

### 2.3 │ Construction of phylogenetic tree

The chloroplast genome sequences of 31 species of Cupressales were downloaded from the NCBI database (Table 1), and three sequence datasets were constructed, respectively. Datasets were as follows: (a) 32 chloroplast genome sequences, (b) 32 sequences of *rbc*L and *mat*K (*rbc*L + *mat*K), and (c) chloroplast genome complete sequences of 14 cypresses and *Juniperus monosperma*. The Clustal W module in MEGA X (Kumar et al., 2018) was used for sequence alignment and correction, and Jmodeltest 2.1.7 was used to predict the optimal nucleotide substitution model. MEGA X was used to construct a neighbor‐joining (NJ) tree. Maximum parsimony (MP) tree was constructed by using PAUP4.0 (Swofford, 2002). RaxmlGUI2 (Stamatakis, 2014) was applied to construct maximum likelihood (ML) tree, and MrBayes3.2.6 (Huelsenbeck & Ronquist, 2001) was used to conduct bayesian inference (BI) tree. Finally, FigTree v1.4.4 was used to draw the phylogenetic tree. The phylogenetic tree for evolutionary analysis was obtained after comparison and correction based on the comprehensive analysis of each tree file and the results of previous studies at the genus level (Gadek et al., 2000; Hao et al., 2016; Lu et al., 2014; Qu, Jin et al., 2017; Qu, Wu et al., 2017).

**TABLE 1:** The information of sample species

Species name	Genbank accession number	Species name	Genbank accession number
Cupressaceae			
*Cupressus chengiana*	NC_034788	*Juniperus monosperma*	NC_024022
*Cu. gigantea*	NC_028155	*J. squamata*	NC_044076
*Cu. jiangeensis*	NC_036939	*Chamaecyparis formosensis*	NC_034943
*Cu. sempervirens*	NC_026296	*Ch. hodginsii*	NC_036996
*Cu. tonkinensis*	NC_039562	*Thuja sutchuenensis*	NC_042176
*Cu. torulosa*	NC_039563	*Thujopsis dolabrata*	KX832628
*Callitropsis funebris*	MT227813	*Callitris rhomboidea*	NC_034940
*Ca. nootkatensis*	KP099642	*Taxodium distichum*	NC_034941
*Ca. vietnamensis*	KX832629	*Glyptostrobus pensilis*	NC_031354
*Hesperocyparis glabra*	KX832624	*Cryptomeria japonica*	NC_010548
*Hesperocyparis lindleyi*	NC_039566	*Sequoia sempervirens*	NC_030372
*H. lusitanica*	MH121051	*Metasequoia glyptostroboides*	NC_027423
*H. benthamii*	NC_039565	*Taiwania flousiana*	NC_021441
*H. arizonica*	NC_039564	*Cunninghamia lanceolata*	NC_021437
*Calocedrus formosana*	NC_023121	*Platycladus orientalis*	KX832626
Taxaceae			
*Amentotaxus argotaenia*	NC_027581		
Sciadopityaceae			
*Sciadopitys verticillata*	NC_029734		

### 2.4 │ Analysis of chloroplast genome structure characteristics

The files containing the complete sequence of the chloroplast genome were imported into OGdraw and Geneious Prime 2020.1.2 (Kearse et al., 2012), and the genomic structure information was generated. MISA (Microsatellite Identification Tool) online website (https://webblast.ipk‐gatersleben.de/misa/index.php?action=1) was employed to predict SSRs. The parameters were set to default settings; SSR motif length corresponding to the minimum number of repetitions is 1–10, 2–6, 3–5, 4–5, 5–5, 6–5. When the distance between two microsatellites is <100 bp, 2 microsatellites were considered to form a composite microsatellite (Beier et al., 2017).

### 2.5 │ Adaptive evolutionary analysis

Geneious Prime 2020.1.2 was used to screen the shared genes of the chloroplast genomes of 32 species, and to extract the coding sequences of the genes. The sequences were aligned and corrected using the ClustalW (Codons) module in MEGA X. Sequence files in.fasta/.fas format were converted to .PML format through DAMBE 7.2.1 (Xia, 2017). Using the codeml program of software PAML 4.9 (Yang, 2007), adaptive evolutionary analysis of common protein‐coding genes was performed based on the ML method in the phylogenetic background of Cupressales.

Three models were used: (a) Branch Model, the variable ω model between branches (Yang, 1998) allows the *ω* value to change in different branches. M0 (One‐ratio) assumes that all evolutionary branches have the same *ω* value. F (Free‐ratio) assumes that each branch has different *ω* values. The M0 and F likelihood ratio test can further determine the positive selection in the branch. Model2 (two‐ratio) assumes that the values of foreground branch and background branch are different. Here, because the cypresses is the research object, the cypresses branch and the ancestor branch of *Cupressus tonkinensis* and *Ca. funebris* were set as the foreground branch, respectively, to examine whether there is any difference in the values of foreground branch and background branch. (b) Site Model, variable ω model between sites (Yang et al., 2000) assumes that different sites have different values of *ω* and there is no difference in different branches of the phylogenetic tree. It mainly includes four pairs of nested models, among them M1a (near neutral) and M2a (positive selection) were for detecting positive selection sites. Site models can be used by BEB (Bayes Empirical Bayes) to identify positively selected sites (Yang et al., 2005). (c) Branch‐Site Model (Yang & dos Reis, 2010). It analyses genes that accept the F model in the Branch model. The alternative hypothesis with cypresses or the ancestor branch of *Cu. tonkinensis* and *Ca. funebris* as the foreground branch and the corresponding null hypothesis with *ω* = 1 were, respectively, tested for likelihood ratio, and the positive selection sites were screened by BEB.

## 3 │ RESULTS

### 3.1 **│** Chloroplast genome structure of *Ca. funebris*

**FIGURE 1:**
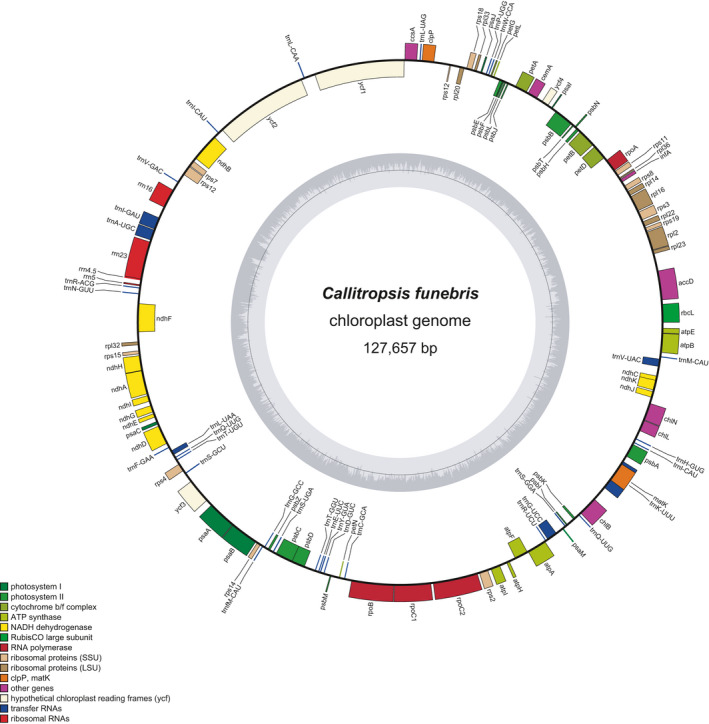
Gene map of the *Callitropsis funebris* chloroplast genome

Raw data of 3.66G were generated, including 12,206,029 raw reads. Standard reads were 150 bp, and 3.37G clean data were obtained after trimming. The complete genome sequence of chloroplast was obtained by assembly, with the total length of 127,657 bp and GC content of 34.7%; and there is no IR sequence in this genome (Figure 1). A total of 115 genes were encoded, including 82 protein‐coding genes, 31 tRNA genes (*trn* I‐CAU and *trn*Q‐UUG are double‐copy genes), and 4 rRNA genes. Twelve genes contained one intron, and two genes (*rps*12, *ycf*3) contained two introns. The Genbank accession number is MT227813.

BLAST search in NCBI showed that *Ca. funebris* had the highest homology with *Cu. tonkinensis* (Query Cover: 100%, Per.ident: 99.87%). The sequence similarity between *Cu. tonkinensis* and *Ca. funebris* is extremely high, and they are sister groups in the phylogenetic tree. Meanwhile, in NCBI *Cu. tonkinensis* Silba is treated as homotypic synonym as *Cu. funebris* (*Ca. funebris*) subsp. *tonkinensis*. Therefore, in the subsequent analysis and discussion, *Cu. tonkinensis* is not regarded as *Cupressus* species, but in the same category with *Ca. funebris*.

### 3.2 │ Phylogenetic relationship of Cupressaceae

**FIGURE 2:**
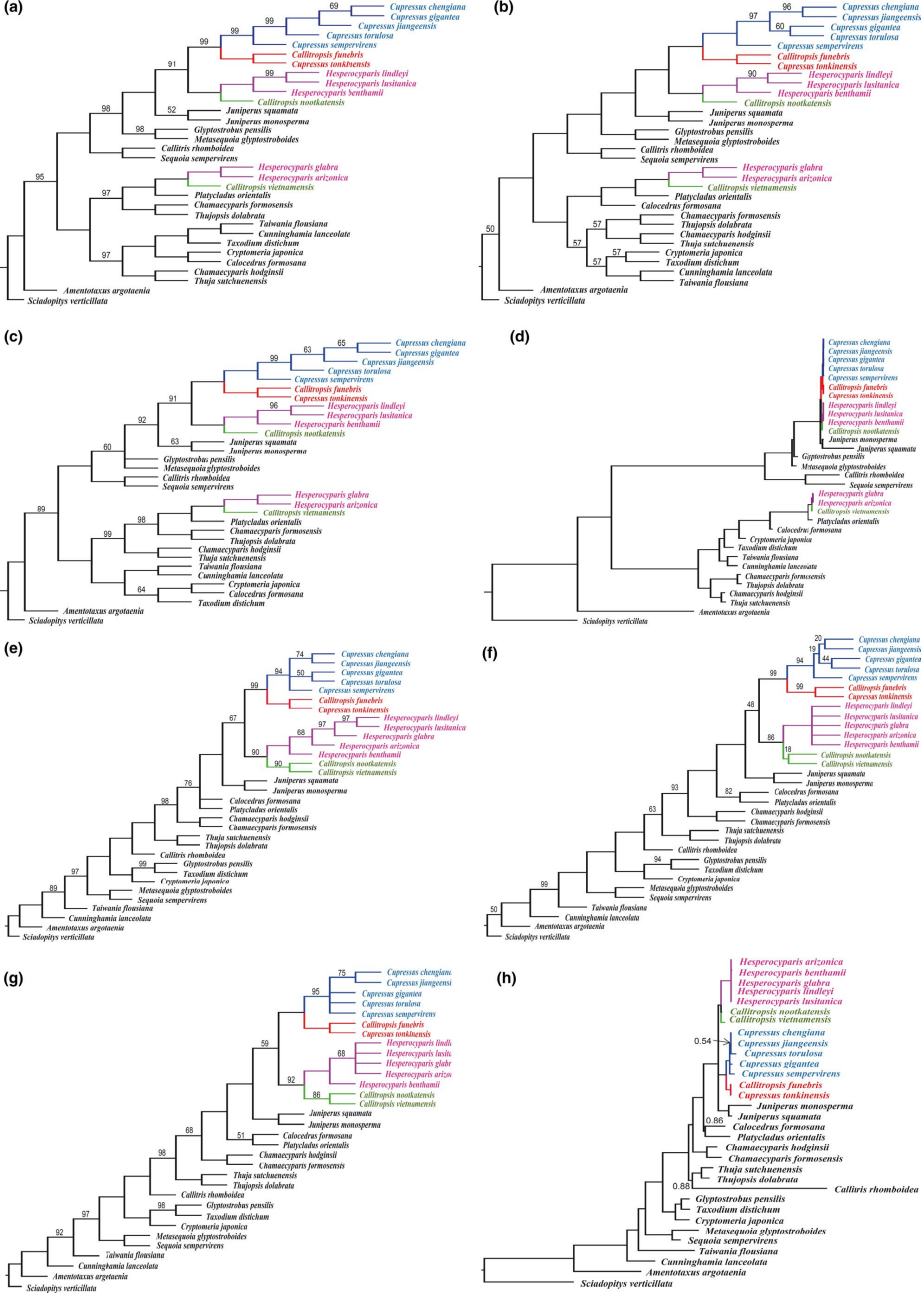
Phylogenetic relationship of Cupressales was construct by four methods and two data sets. (a–d) based on the complete chloroplast genome. (e–h) based on the series data of *rbc*L and *mat*K (*rbc*L + *mat*K). (a–d) and (e, f) correspond to NJ tree, MP tree, ML tree, and Bayes tree, respectively. The number on the branch is the bootstrap support value. Blue branch: *Cupressus* (except *Cu. tonkinensis*). Red branch: *Ca. funebris* and *Cu. tonkinensis*. Purple branches: *Hesperocyparis*. Green branch: *Ca. nootkatensis* and *Ca. vietnamensis*

Taking *Sciadopitys verticillata* as the outgroup, phylogenetic analysis based on the complete sequence of the chloroplast genome showed that the relationship among Cupressaceae was chaotic (Figure 2a–d). Like the *Chamaecyparis*, the species branches of *Hesperocyparis* were not clustered together. And the support among the branches was low. Phylogenetic analysis based on *rbc*L + *mat*K showed clearly the relationship of Cupressaceae (Figure 2e–h). Among them, cypresses form a monophyletic branch and the monophyletic branch of *Ca. nootkatensis* and *Ca. vietnamensis* is sister group to *Hesperocyparis*. Also, branch relationships among other genera have higher support. It should be noted that there is a paraphyletic relationship between *Cupressus* and *Hesperocyparis*; the relationship between the related species is difficult to determine. All phylogenetic trees consistently showed that *Cu. tonkinensis* and *Ca. funebris* are sister branches to each other, and the monophyletic branch formed by the two are sister group to the *Cupressus*.

### 3.3 │ Chloroplast genome structure of cypresses

Chloroplast genome size of cypresses is 127,005 bp–129,150 bp, GC content is 34.6%–34.7%, and the number of genes, protein‐coding genes, and tRNA coding genes is highly consistent, 119, 82, and 33, respectively. The difference is that the genome size of *Cupressus* is between 128,151 and 129,150 bp, and that of *Hesperocyparis* is between 127,005 and 127,158 bp. Numerically, *Ca. funebris* is closer to *Cupressus*. *Ca. nootkatensis* and *Ca. vietnamensis* are closer to *Hesperocyparis* (Table 2).

**TABLE 2:** Genome structure characteristics of cypresses and *Chamaecyparis*

Species name	Genome size/bp	GC content	Genes	Protein‐coding genes	tRNA
*Cu. chengiana*	128,151	34.70%	119	82	33
*Cu. gigantea*	128,244	34.70%	119	82	33
*Cu. jiangeensis*	128,286	34.70%	119	82	33
*Cu. sempervirens*	129,150	34.60%	119	82	33
*Cu. torulosa*	128,322	34.60%	119	82	33
*Cu. tonkinensis*	127,835	34.70%	119	82	33
*Ca. funebris*	127,657	34.70%	119	82	33
*Ca. nootkatensis*	127,150	34.70%	119	82	33
*Ca. vietnamensis*	127,479	34.70%	119	82	33
*H. glabra*	127,064	34.70%	119	82	33
*H. lindleyi*	127,005	34.70%	119	82	33
*H. lusitanica*	127,113	34.70%	119	82	33
*H. benthamii*	127,007	34.70%	119	82	33
*H. arizonica*	127,158	34.70%	119	82	33
*Ch. formosensis*	127,211	35.00%	121	85	32
*Ch. hodginsii*	127,777	35.00%	120	83	33

**FIGURE 3:**
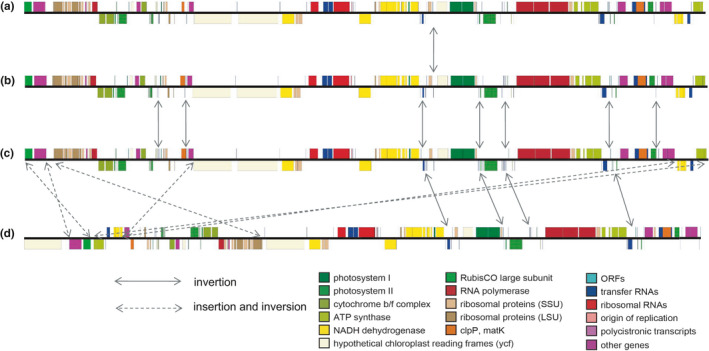
Gene arrangement of chloroplast genomes of cypresses and *Chamaecyparis*. (a) *Cu. chengiana*, *Cu. gigantea*, *Cu. jiangeensis*, *Cu. sempervirens*, and *Ca. nootkatensis*. (b) *Cu. torulosa*, *H. lusitanica*, *H. glabra*, *H. lindleyi*, *H. benthamii*, *H. arizonica*, *Ca. vietnamensis*. (c) *Ca. funebris*, *Cu. tonkinensis*. (d) *Ch. formosensis*, *Ch. Hodginsii*

Genes of the cypress species are in the same order and are divided into three types according to the location of the genes (Figure 3). Type A includes *Cupressus* and *Ca. nootkatensis*, type B includes *Hesperocyparis* and *Ca. vietnamensis*, and type C includes *Ca. funebris* and *Cu. tonkinensis*. Only one gene is inverted between type A and type B, and there are 11 genes that are inverted between type C and type A, as well as between type C and type B. Of note, type D contains *Chamaecyparis*. At least 3 insertion‐inversion events and inversion of multiple genes occur between type D and type C.

### 3.4 │ Phylogenetic relationship of cypresses

**FIGURE 4:**
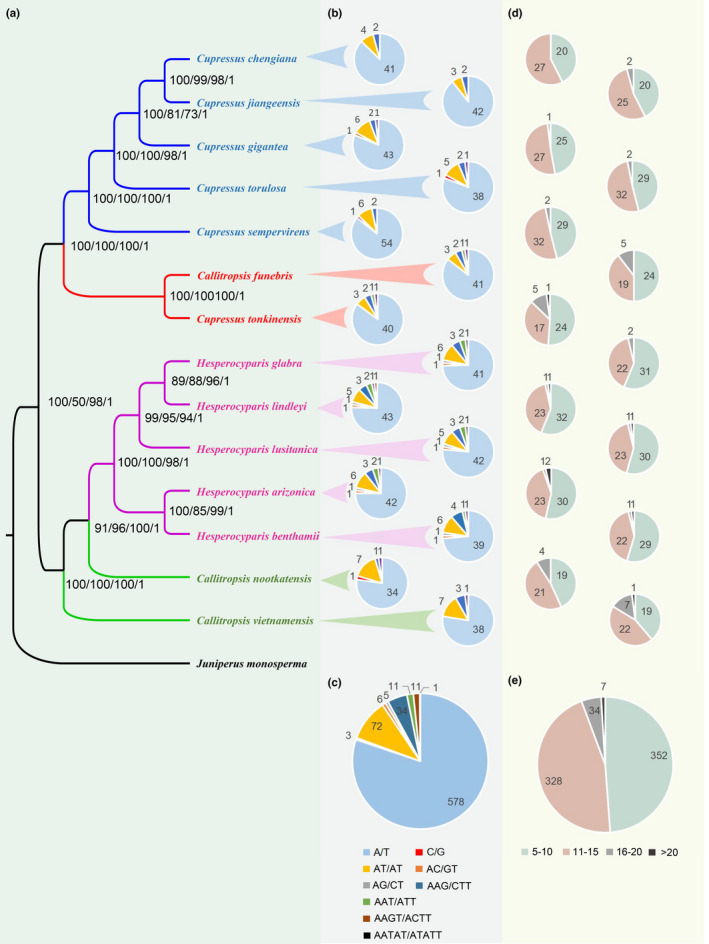
Phylogenetic relationship of cypresses and SSRs analysis. (a) With the complete sequence of chloroplast genome, the phylogenetic relationship of cypresses was constructed based on NJ, MP, ML, and BAYES, and the number of node locations, respectively, represents the branch support corresponding to the four methods. (b) the number and type of the motifs of each species. (c) the number and type of motifs of all species. (d) distribution of the number of repetitions in each species. (e) distribution of the number of repetitions for all species

In order to further determine the phylogenetic relationship of cypresses, the complete chloroplast genome sequences of 14 cypresses and *J. monosperma* were analyzed. The genes of all 15 species are in the same order on the genome, with *J. monosperma* as the outgroup. The results showed that the phylogenetic relationship obtained by the four methods was consistent. The monophyletic branch formed by *Ca. funebris* and *Cu. tonkinensis* is the sister group of the remaining *Cupressus*. *Ca. nootkatensis* and *Ca. vietnamensis* were in turn nested at the base of the monophyletic group *Hesperocyparis*. These branch relationships are strongly supported (Figure 4a).

### 3.5 │ SSR analysis of cypresses

All cypresses detected a total of 721 SSRs, including a total of 5 repeat types and 8 types of repeat motifs (Figure 4c and Appendix S1). The number of mononucleotide repeats is the largest, totaling 581 (80.6%), of which A/T repeat motif accounts for 99.4%. Dinucleotide repeats contain 3 types of repeat motifs (AT/AT, AC/GT, AG/CT), in total 83, of which AT/AT accounts for 86.75%. Trinucleotide repeats have two kinds of repeat motifs, a total of 45, mainly AAG/CTT (75.56%). Both tetranucleotide repeats and pentanucleotide repeats contain only one kind of repeat motif. Pentanucleotide repeats were only found in *H. lindleyi*. Overall, SSRs are dominated by AT base (A/T, AT/AT, AAT/ATT, AATAT/ATATT), accounting for 91.82% of the total.

**FIGURE 5:**
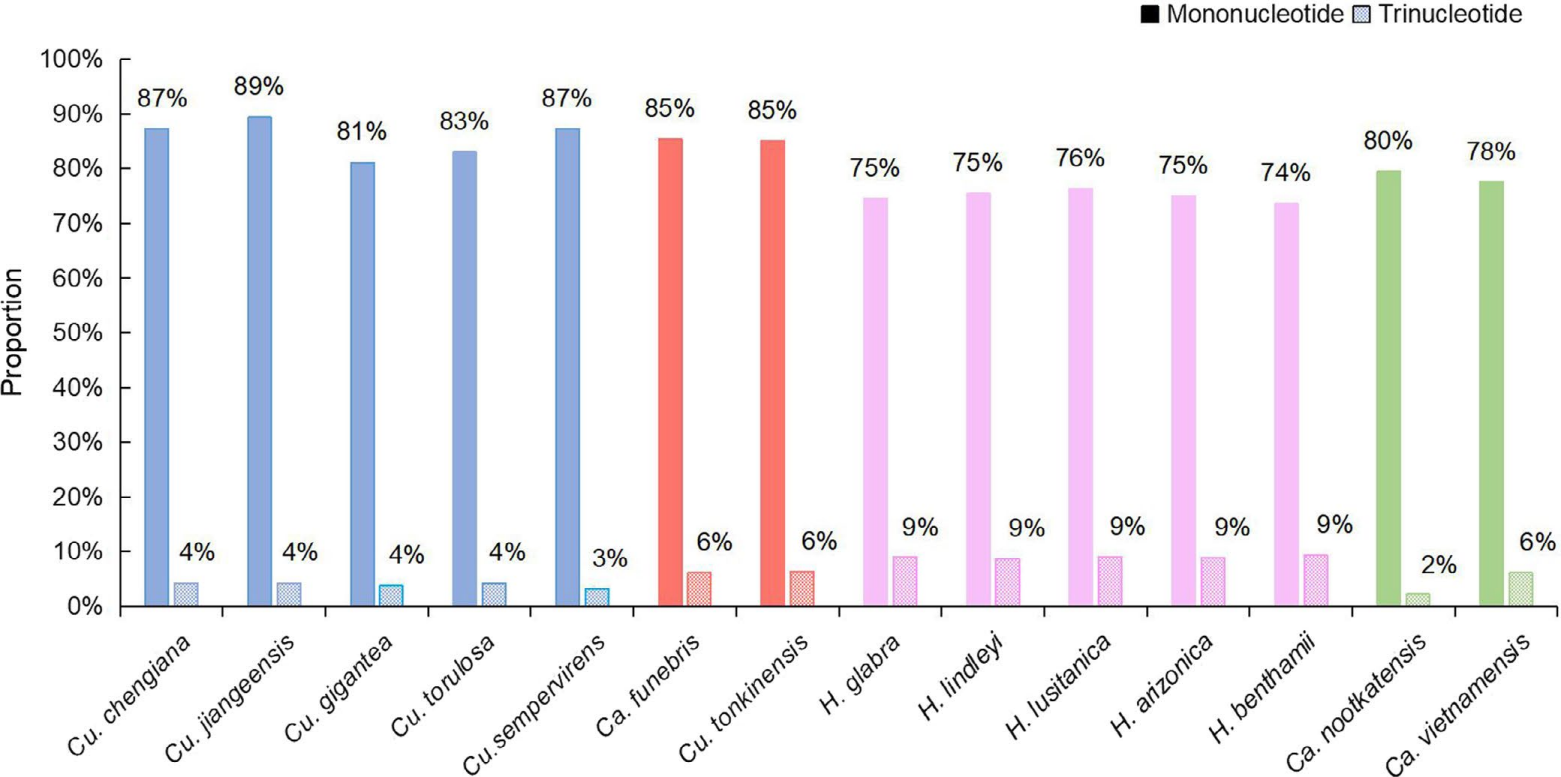
The proportion of mononucleotide repeats and trinucleotide repeats in each species

Different species have different types and contents of motifs. Of more obvious features, AG/CT exists only in the *Hesperocyparis*. AAT/ATT is found in *Ca. funebries*, *Cu. tonkinensis* and *Hesperocyparis*, not in *Callitropsis* and *Cupressus*. *Hesperocyparis* has the most abundant types of motifs, except for *H. lindleyi*, which contains eight types of repeat motifs, and the remaining four species contain seven types, respectively (Figure 4b and Appendix S1). In terms of content, due to the scarcity of G/C motif, the proportion of A/T motif is close to that of mononucleotide, and the proportions of mononucleotide repeats were counted in each species (Figure 5): *Cupressus* (81%–89%), *Hesperocyparis* (74%–76%), *Ca. funebries*, *Ca. nootkatensis* and *Ca. vietnamensis*: 85%, 80%, 78%. The proportion of trinucleotide repeats in various species is: *Cupressus* (3.2%–4.3%), *Ca. funebries* and *Cu. tonkinensis* (6.3%, 6.4%), *Hesperocyparis* (8.9%–9.4%), *Ca. nootkatensis* and *Ca. vietnamensis* (2.3%, 6.1%).

The number of repetitions is distributed between 5 and 47, which is divided into four intervals (Figure 4e). The number of 5–10 repetitions is the most (352, 48.8%), and the number of repetitions >20 is the least (7, 0.9%). The distribution of the number of repetitions in different groups has lineage characteristics (Figure 4d, Table 3). The number of 5–10 repetitions is the highest in *Hesperocyparis* (53.5%–56.1%), the number of 11–15 repeats is the highest in the *Cupressus* (50.8%–57.4%), and the number of 16–20 repeats is the highest in the *Callitropsis* (9%–14%).

**TABLE 3:** The proportion of the number of repetitions in different groups

Repeat number	*Cupressus*	*Ca. funebries*	*Cupressus tonkinensis*	*Hesperocyparis*	*Ca. nootkatensis*	*Ca. vietnamensis*
5–10	42.6−47.2%	50%	51.1%	53.5−56.1%	43.1%	38.8%
11–15	50.8−57.4%	39.6%	36.2%	40−41.8%	47.7%	44.9%
16–20	0−4.3%	10.4%	10.6%	1.8−3.6%	9.1%	14.3%
>20	0%	0%	2%	0−4%	0%	2%

**FIGURE 6:**
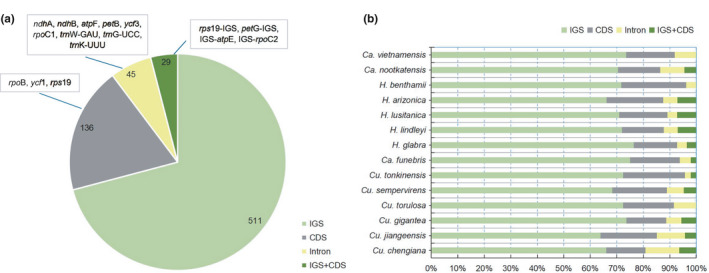
The position distribution of the repeated sequence on the genome. (a) The distribution of all the repeating sequences. Most distributed in the intergenic spacer (IGS) region (70.9%), and at least 4% are distributed across structural regions (IGS + CDS). (b) The position distribution of the repeated sequence of each species

Location of SSRs on the chloroplast genome was examined. 70.9% were located in the intergenic spacer region (IGS), and 18.9% were located in coding sequence region (CDS) (Figure 6a). The SSRs of each species were mainly distributed in the IGS region (63.8%–76.4%) and less in IGS + CDS (Cross‐structural) region (Figure 6b). Of the 29 SSRs distributed in IGS + CDS, 19 were located in *rps*19‐IGS. The SSRs located in IGS‐*atp*E occur only in *Cupressus* and *Ca. funebries*, whereas the SSRs located in *pet*G‐IGS occur only in *Hesperocyparis*. There is at least one AAG/CTT distributed in the CDS region of each species, and this region is limited to *ycf*1or *rpo*B. Specifically, there is only one located at *ycf*1 in *Ca*. *nootkatensis*, only one located at *rpo*B in *Ca. vietnamensis*, and there are 2–3 located at *ycf*1 and *rpo*B in the remaining species (Appendix S1).

### 3.6 │ Analysis on adaptive evolution of Cupressales

**FIGURE 7:**
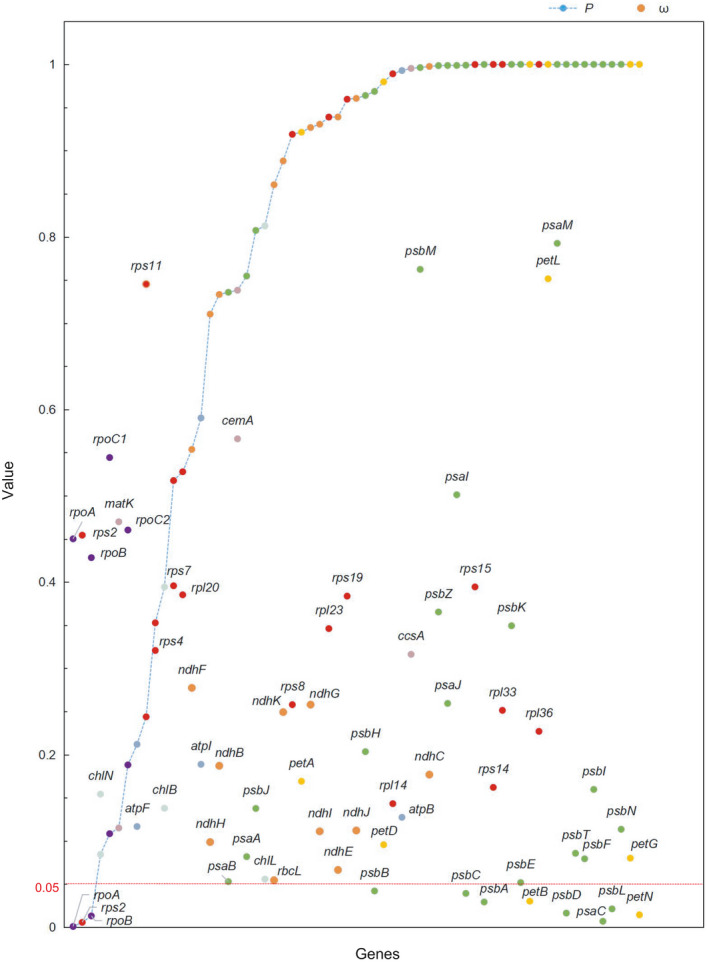
Under the Branch model, the likelihood ratio of M0 and F model was tested to get the *p* value and *ω* in M0. Three genes (*rps*2, *atp*B, and *rpo*A) accepted the F model (*p* < 0.05)

**FIGURE 8:**
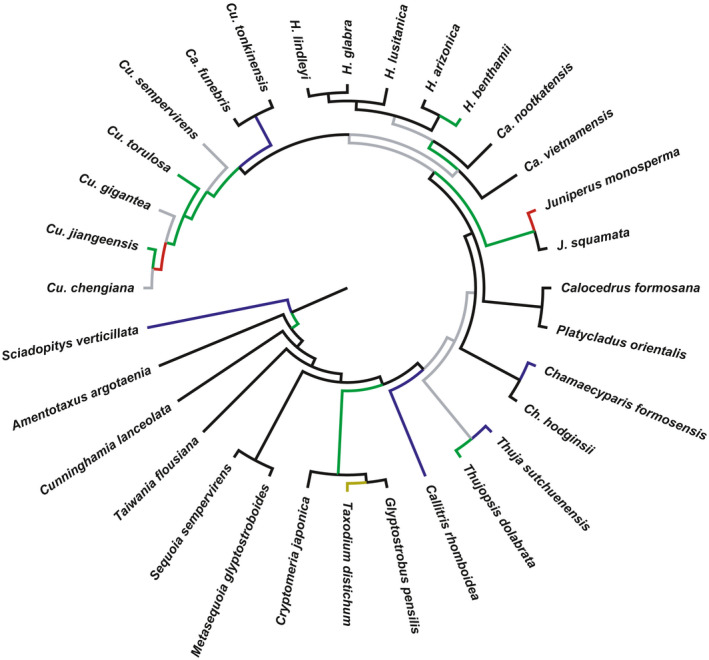
Phylogenetic relationship of Cupressales. Its also a tree file in the PAML analysis. Branches with different colors represent branches with *ω* > 1 in the three genes in the Branch model that accept the F model. Blue: *rps*2, Red: *atp*B, Green: *rpo*A. Gray: *rpo*A and *rps*2 shared. Yellow: three genes shared. The positive selective branches appear mostly in the middle and late stages of the evolution of Cupressaceae

A total of 63 common genes were selected (Table 4), including 43 photosynthetic system genes, 17 genetic expression genes, and 3 miscellaneous protein genes. Based on the phylogenetic relationship of Cupressales (Figure 2) and cypresses (Figure 4a) obtained above, the phylogenetic tree of Cupressales was manually adjusted (Figure 8). Under the Branch model, the likelihood ratio test results of M0 and F showed that 60 genes accepted the M0 model (*p* > .05) and 3 genes (*rps*2, *atp*B, *rpo*A) accepted the F model (*p* < .05) (Figure 7). For *rps*2, *atp*B and *rpo*A, there were 15, 3, and 21 branches with the *ω* value >1, respectively, and the specific branch positions were shown in Figure 8. In the likelihood ratio test of Model2 and M0 with cypresses as the foreground, there were three genes with significant differences: *rps*19 (*ω*_backgroud_ = 0.38377, *ω*_foreground_ = 0.0001), *rpl*20 (*ω*_backgroud_ = 0.38526, *ω*_foreground_ = 0.0001), and *rpo*C2 (*ω*_backgroud_ = 0.46030, *ω*_foreground_ = 0.47076). The likelihood ratio test results of Model2 and M0 with the ancestral branches of *Ca. funebries* and *Cu. tonkinensis* as the foreground branch showed that only the *rpo*C2 had a significant difference (*ω*_backgroud_ = 0.46030 and *ω*_foreground_ = 0.47076, *p* < .05). In the Site model, based on the likelihood ratio test results of M2a and M1a (Appendix S2), there were 17 genes with significant differences (*p* < .05). With posterior probability *p* > 99% as the standard, positive selection sites were detected in 10 genes (Table 5). The Branch‐Site model detected an amino acid site under positive selection in the *rpo*A gene with *Ca. funebries* and *Cu. tonkinensis* as foreground branches (Appendix S3).

**TABLE 4:** Common genes of sample species for molecular evolutionary analysis

Gene type		Gene name
Genes for photosynthesis	Photosystem Ⅰ	*psa*A *psa*B *psa*C *psa*I *psa*J *psa*M
	Photosystem Ⅱ	*psb*A *psb*B *psb*C *psb*D *psb*E *psb*F *psb*H *psb*I *psb*J *psb*K
		*psb*L *psb*M *psb*N *psb*T *psb*Z
	Cytochrome	*pet*A *pet*B *pet*D *pet*G *pet*L *pet*N
	ATP synthase	*atp*B *atp*F *atp*I
	RubiscoCO large subunit	*rbc*L
	NADH dehydrogenase	*ndh*B *ndh*C *ndh*E *ndh*F *ndh*G *ndh*H *ndh*I *ndh*J *ndh*K
	Chlorophyll biosynthesis	*chl*B *chl*L *chl*N
Genetic system	Ribosomal proteins (LSU)	*rpl*14 *rpl*20 *rpl*23 *rpl*33 *rpl*36
genes	Ribosomal proteins (SSU)	*rps*2 *rps*4 *rps*7 *rps*8 *rps*11 *rps*14 *rps*15 *rps*19
	RNA polymerase	*rpo*A *rpo*B *rpo*C1 *rpo*C2
Others genes	Maturase	*mat*K
	Envelop membrane protein	*cem*A
	c‐type cytochrome synthesis	*ccs*A

**TABLE 5:** The positive selective amino acid sites were selected from the positive selective genes. (Take the amino acid sequence of *Ca. nootkatensis* as the reference sequence)

Gene	Positively selected amino acid sites
*rpo*A	165S, 252S, 331G
*rpo*B	51T, 94C, 99Q, 214Y, 217K, 248E, 371L, 376T
*rpo*C1	41Q, 75P, 76M, 84Y, 166E, 178D, 234L, 240T, 241Q, 243L, 244N, 245Q, 246D, 247S, 248P, 254D, 256I, 331N, 338W
*rpo*C2	9P, 464P, 469E, 644L, 1126P, 1135I, 1147L, 1150Q, 1156L, 1159L, 1160V, 1162I, 1167K, 1168R
*rps*7	67E, 93E
*rps*11	109K
*rpl*20	116L
*psb*M	30K
*cem*A	90K, 129K
*ccs*A	205R

## 4 │ DISCUSSION

### 4.1 **│** *Callitropsis funebris* is more closely relate to the *Cupressus* than *Callitropsis*

Phylogenetic relationships among *Juniperus* and cypresses of Cupressaceae are controversial. Our result showed the 8 topological structures constructed for 32 species are incongruent. Particularly, the phylogenetic relationships from complete chloroplast genome were chaotic (Figure 2a–d). Specifically, the *Hesperocyparis* and *Chamaecyparis* did not form a monophyletic branch, respectively. The possible reason is due to the multiple rearrangements of the genome (especially inversions [Wu & Chaw, 2016]) during the evolution of Cupressales. So it is difficult to align during the alignment process, thereby losing most of the informative sites and causing phylogenetic relationship chaos. In short, genome rearrangement has an impact on sequence alignment analysis. However, we observe that the monophyletic branch of *Ca. funebris* and *Cu. tonkinensis* in the four topological structures is always sister groups with *Cupressus*.

*rbc*L and *mat*K are used as DNA Barcodes (Hollingsworth et al., 2011), and the obtained phylogenetic relationship has a high degree of recognition. But it is uncertain to resolve deeper branching relationships due to too few sequence differences (Tonti‐Filippini et al., 2017). As it appears in the results, there is a parallel relationship among related species (Figure 2e–h). However, the phylogenetic relationship between genera is very clear and consistent with the phylogenetic relationship constructed from nuclear genes (Lu et al., 2014) or plastid genomes (Gadek et al., 2000; Hao et al., 2016; Qu, Jin, et al., 2017; Qu, Wu, et al., 2017). Also, all trees show that the monophyletic branch of *Ca. funebris* and *Cu. tonkinensis* is always sister groups with *Cupressus*. It should be noted that, in addition to the BI tree, the other three trees all show that cypresses is a monophyletic group, but the support is not high (NJ:67, MP:48, ML:59). This also shows the complexity of cypresses and *Juniperus* (Zhu et al., 2018).

### 4.2 │The complexity of cypresses (especially *Callitropsis*)

In this research, in order to further determine the phylogenetic relationship of the cypresses, the complete sequence of 15 chloroplast genomes with the same gene order was selected. All trees highly support *Hesperocyparis* and *Cupressus* to form monophyletic branches, respectively (Figure 4a), which is consistent with the results of Mao et al. (2010) based on nuclear genome sequence and chloroplast genome sequence. Consistent with Figure 2, the monophyletic branch formed by *Ca. funebris* and *Cu. tonkinensis* presents as the sister group of *Cupressus*.

Among them, the most controversial is *Callitropsis*. Farjon et al. (2002) classified a new cypress species from Vietnam into a new genus—*Xanthocyparis*, and a particular cypress species (*Cupressus nootkatensis*) into the new genus. Little (2006) speculated that the *Cupressus* is paraphyletic, and the New World cypresses diverged from a clade that later produced *Juniperus*. He assigned the New World cypresses and *Xanthocyparis nootkatensis and Xanthocyparis vietnamensis* to the genus *Callitropsis*. In subsequent studies, the phylogenetic relationships constructed from nuclear genome and chloroplast genome data showed that *Ca. nootkatensis* and *Ca. vietnamensis* are nested in turn at the base of the monophyletic group *Hesperocyparis* (Mao et al., 2010; Terry et al., 2012). This is consistent with the results of this study, indicating that *Callitropsis* under the existing classification system is not a monophyletic group. Because of this, recent research supports the division of 4 genera in cypresses, namely *Callitropsis*, *Cupressus*, *Hesperocyparis,* and *Xanthocyparis* (Zhu et al., 2018).

A common factor that interferes with phylogenetic analysis is hybridization events, especially when using cytoplasmic loci. If recombination occurs between different plastid haplotypes, the impact will be more serious (Wolfe & Randle, 2004). In conifers, hybridization may lead to chloroplast capture, nuclear introgression, and phylogenetic inconsistencies between the nuclear and plastid genomes (Peng & Wang, 2008; Sullivan et al., 2017; Xiang et al., 2015). Studies have found that uniparental inheritance of mitochondrial and chloroplast genomes (maternal‐to‐paternal and vice versa) is often reversed (Whittle & Johnston, 2002), and genetic leakage has been observed in many Cupressaceae species and other seed plants (Wagner et al., 1991; Weihe et al., 2009). Therefore, cypresses may have undergone some degree of network evolution. In general, the research has enriched the plastid genomics research of cypresses, and we need to combine more effective data sets in future exploration.

### 4.3 │ The unique chloroplast genomic structure of *Ca. funebris*

During the evolution of plants, the chloroplast genome often undergoes some changes, including rearrangement (Guisinger et al., 2010; Wolf et al., 2011; Wu et al., 2007) and gene loss (Braukmann et al., 2009; Delannoy et al., 2011). These changes occur independently and are often unique to specific taxa (Wolf et al., 2010). During the evolution from *Chamaecyparis* to cypresses, the chloroplast genome structure has at least 3 insertions and inversions (Figure 3). The structural characteristics of cypresses' chloroplast genome show high consistency. Only one gene between *Cupressus* and *Hesperocyparis* is inverted. *Ca. nootkatensis* and *Ca. vietnamensis* are the same as *Cupressus* and *Hesperocyparis*, respectively, showing the particularity of *Callitropsis*. In particular, compared with *Cupressus* and *Hesperocyparis*, several genes of *Ca. funebris* were inverted, showing the uniqueness of *Ca. funebris*. Existing studies have shown that in Podocarpaceae and Taxaceae, the number of inversions between each genera differs by 6 times (Wu & Chaw, 2016). Similarly, *Ca. nootkatensis* and *Ca. vietnamensis* should belong to different genera according to the inversion. This seems to be consistent with the views of previous studies (Zhu et al., 2018). And *Ca. funebris* should be considered as an independent genus. Moreover, from the analysis of chloroplast genome (GC content, gene number, and genome structure), the division of *Ca. funebris* and *Chamaecyparis* is not disputed.

### 4.4 │ The distribution pattern of SSRs is beneficial to the phylogenetic relationship study

SSRs are dominated by A/T bases, and very few G/C were found and are mainly located in the IGS region. This is consistent with the existing chloroplast SSR report (Gui et al., 2020; Li et al., 2017). They show lineage specificity on the type and proportion of repeat motif and the distribution of repeat times. *Cupressus* and *Hesperocyparis* show highly uniform characteristics, respectively, while *Callitropsis* differs from them. There are differences between *Ca. funebris* and other two species in *Callitropsis*. In the proportion of A/T motif and mononucleotide repeats, *Ca. funebris* is closer to the characteristics of *Cupressus* (Figure 4b). In the SSR multidirectional comparison, *Cupressus* and *Callitropsis* have notable differences. Overall, SSRs are abundant in cypresses and can be used for intergenus identification, as well as to detect genetic diversity at the population and interspecies level.

### 4.5 │ Adaptation changes occurred during the evolution of Cupressales

During the evolution of Cupressales, positive selection branches were detected in three genes, and the positive selection effect of these three genes occurred in the middle and early stages of Cupressales evolution. There are two genes with an obvious difference in selection pressure detected between cypresses and other branches, indicating that the two genes are experienced to strong negative selection effect in the cypresses branch.

To better understand the evolutionary history of Cupressales, the analysis of its genetic diversity and adaptive evolution is essential. Positively selected genes play an important role in the adaption to various environments. Ten genes with positively selected sites were detected in the Site model, seven genes belonged to genetic system gene (*rpo*‐, *rps*7, *rps*11, *rpl*20), 1 photosystem gene (*psb*M), and two other genes (*cem*A, *ccs*A). *Rpo*‐ gene encodes RNA polymerase, the most critical enzyme in transcription. *Rps*7, *rps*11, and *rpl*20 encode ribosome 30S small subunit S7, S11 protein, and 50S large subunit L20 protein, respectively, all of which are involved in protein synthesis. *Psb*M is one of the components of the core complex of photosystem II. *cem*A encodes a chloroplast envelope membrane protein (Sasaki et al., 1993) and is inferred to indirectly influence CO_2_ uptake in plastid (Rolland et al., 1997). The *ccs*A gene encodes a protein required for heme attachment to c‐type cytochromes (Merchant, 1996).

Understanding the patterns of divergence and adaptation among the members of a specific phylogenetic clade can offer important clues about the forces driving its evolution (Li et al., 2019). In this study, we detected that only the *rpo*A gene had undergone positive selection in the *Ca. funebris* branch, and only one positively selected site was detected. This may be due to plants having multiple strategies to adapt to the environment, and the adaptive modification of other abiotic stress‐targeted genes in the nucleus is sufficient to maintain photosynthesis homeostasis. As a result, adaptive evolution of chloroplast‐encoded genes is not required (Li et al., 2019).

All these genes might play important roles when founder effects occur in populations, both changes in selection pressure and genetic drift may result in the rapid shift of these genes to a new coadapted combination. The results provide an insight into adaptive evolution of Cupressales and a basis for further clarifying the chloroplast genetic characteristics.

## 5 │ CONCLUSIONS

Morphological‐based research has a limited role in diagnosing the relationship between orders or classes of any group of plants, especially for intermediate types such as cypresses. The analysis from molecular data can provide strong support and insights for its phylogenetic analysis. Different from its morphological characteristics, the analysis results based on the phylogenetic relationship and genomic characteristics (SSRs, etc.) of the complete chloroplast genome sequences support that the monophyletic branch of *Ca. funebris* and *Cu. tonkinensis* is a sister branch of *Cupressus* (or as the base group of *Cupressus*). And *Callitropsis* is not a monophyletic group, *Ca. nootkatensis* and *Ca. vietnamensis* are nested in turn at the base of the monophyletic group *Hesperocyparis*. At the same time, *Ca. funebris* and *Chamaecyparis* are two different groups. During the evolution of Cupressales, multiple genes were detected to experience positive selection, suggesting that they may have undergone adaptive changes. One problem is that the data for constructing the topological structure only come from the chloroplast genome, which is far from enough. It is necessary to combine the nuclear genome or mitochondrial genome data, which will be more conducive to understanding their evolutionary process.

## CONFLICT OF INTERESTS

The authors declare no conflict of interest.

## AUTHOR CONTRIBUTION

**jingyao Ping:** Conceptualization (equal); Data curation (equal); Writing‐original draft (equal). **Peipei Feng:** Data curation (equal). **Jinye Li:** Data curation (equal); Formal analysis (equal). **Rongjing Zhang:** Resources (equal). **Ying‐juan Su:** Writing‐review & editing (equal). **Ting Wang:** Conceptualization (equal); Funding acquisition (equal); Writing‐review & editing (equal).

## ETHICS STATEMENT

The research does not involve any ethical issues.

## Supporting information

Appendix S1

Appendix S2

Appendix S3

## Data Availability

Data source is NCBI database: https://www.ncbi.nlm.nih.gov/nuccore/NC_034788,NC_028155,NC_036939,NC_026296,NC_039562,NC_039563,KP099642,KX832629,KX832624,NC_039566,MH121051,NC_039565,NC_039564,NC_023121,NC_024022,NC_044076,NC_034943,NC_036996,NC_042176,KX832628,NC_034940,NC_034941,NC_031354,NC_010548,NC_030372,NC_027423,NC_021441,NC_021437,KX832626,NC_027581,NC_029734,MT227813.
